# Adaptations in Basal and Hypothalamic–Pituitary–Adrenal-Activated Deoxycorticosterone Responses Following Ethanol Self-administration in Cynomolgus Monkeys

**DOI:** 10.3389/fendo.2017.00019

**Published:** 2017-02-06

**Authors:** Vanessa A. Jimenez, Patrizia Porcu, A. Leslie Morrow, Kathleen A. Grant

**Affiliations:** ^1^Oregon National Primate Research Center, Division of Neuroscience, Beaverton, OR, USA; ^2^Department of Behavioral Neuroscience, Oregon Health & Science University, Portland, OR, USA; ^3^Bowles Center for Alcohol Studies, University of North Carolina at Chapel Hill, Chapel Hill, NC, USA; ^4^Neuroscience Institute, National Research Council of Italy (CNR), Cagliari, Italy

**Keywords:** deoxycorticosterone, hypothalamic–pituitary–adrenal axis, cynomolgus monkey, ethanol, schedule induction of ethanol, ethanol self-administration

## Abstract

Acute ethanol activates the hypothalamic–pituitary–adrenal (HPA) axis, while long-term exposure results in a blunted neuroendocrine state, particularly with regards to the primary endpoint, cortisol, the primary glucocorticoid produced in the adrenal cortex. However, it is unknown if this dampened neuroendocrine status also influences other adrenocortical steroids. Plasma concentration of the mineralocorticoid and neuroactive steroid precursor deoxycorticosterone (DOC) is altered by pharmacological challenges of the HPA axis in cynomolgus monkeys. The present study investigated HPA axis regulation of circulating DOC concentration over the course of ethanol (4% w/v) induction and self-administration in non-human primates (*Macaca fasciculata, n* = 10). Plasma DOC, measured by radioimmunoassay, was compared at baseline (ethanol naïve), during schedule-induced polydipsia, and following 6-months of 22 h/day access to ethanol and water. The schedule induction of ethanol drinking did not alter basal DOC levels but selectively dampened the DOC response to pharmacological challenges aimed at the anterior pituitary (ovine corticotrophin-releasing hormone) and adrenal gland (post-dexamethasone adrenocorticotropin hormone), while pharmacological inhibition of central opioid receptors with naloxone greatly enhanced the DOC response during induction. Following 6 months of ethanol self-administration, basal DOC levels were increased more than twofold, while responses to each of the challenges normalized somewhat but remained significantly different than baseline. These data show that HPA axis modulation of the neuroactive steroid precursor DOC is markedly altered by the schedule induction of ethanol drinking and long-term voluntary ethanol self-administration. The consequences of chronic ethanol consumption on HPA axis regulation of DOC point toward allostatic modification of hypothalamic and adrenal function.

## Introduction

Endogenous stress systems are sensitive to pharmacological doses of ethanol as well as the conditions under which ethanol is made available to the organism. The hormonal response to stress in alcoholics involves all three components of the hypothalamic–pituitary–adrenal (HPA) axis and depends on whether they are intoxicated, actively drinking but not intoxicated, in acute withdrawal, or in abstinence. Intoxication (1) and withdrawal (2–4) are associated with increased cortisol secretion in alcohol-dependent patients, which may depend on whether withdrawal is abrupt (5). In non-intoxicated, non-abstinent alcoholics, adrenocorticotropin hormone (ACTH) response to corticotrophin-releasing hormone (CRH) was blunted relative to controls and the time to peak ACTH was delayed in half the subjects (6). Cortisol response to ACTH was blunted, indicative of adrenal insufficiency (6), similar to blunted salivary cortisol during a stress imagery procedure (7). Altered pituitary response to CRH and adrenocortical response to ACTH in alcoholics may recover with abstinence (4, 8). Other studies have found abstinent alcoholics have greater basal ACTH (9), lower levels of the cortisol precursor 11-deoxycortisol and a reduced cortisol response to exogenous ACTH administered after dexamethasone (10). Furthermore, ACTH [early abstinence (11, 12)] and cortisol after pituitary stimulation by CRH are lower in abstinent alcoholics [1-month abstinence (9)]. Blocking inhibitory opioid input to CRH neurons in the hypothalamus with the μ-opioid receptor antagonist naloxone elevated HPA response (13), and this effect is decreased in early (11) but not later (9) abstinence as measured by ACTH. Finally, abstinent alcoholic men (10), but not women (14), had lower dexamethasone (8 mg, i.v.) suppression of ACTH and cortisol compared to controls. Overall these data suggest adaptations during chronic ethanol intoxication in state dependent and likely occur at each level of the HPA axis.

The HPA axis response to ethanol has been suggested as a risk factor for developing alcoholism, and acute, low doses of ethanol suppress cortisol more among alcohol-drinking sons of alcoholics than controls (15). Human studies have the potential confounds of self-selection (non-randomized) drinking status, use of other substances and comorbidities associated with early-onset alcohol drinking and dependence such as life stress, anxiety, and depression. Studies in rodents are limited, as rodents do not have adrenal anatomy and physiology similar to human and non-human primates. Specifically, rodents do not have a 3-layer adrenal cortex, as the zona reticularis is absent (16). Biochemically, there are key differences in steroidogenesis between rodents and primates. For example, human cytochrome P450c17 enzyme rapidly converts 17α-hydroxypregnenolone to dehydroepiandrosterone (DHEA), the principal source of androgens in humans. In contrast, rodent P450c17 efficiently converts 17α-hydroxyprogesterone to androstenedione, the main source of androgens in rodents (17). Furthermore, adult rats have low activity of 5β-reductase (18), and 5β-reduced neuroactive metabolites are low or undetectable basally (19), indicating species differences in the balance of neuroactive metabolites. In addition, non-human primates share with humans the propensity to self-administer large quantities of ethanol over months and years, with similar absorption and metabolism (20, 21). These differences suggest monkeys are excellent models for studying the interaction between steroid and neuroactive steroid metabolites and ethanol self-administration.

Deoxycorticosterone (DOC) is a mineralocorticoid synthesized in the zona fasciculata and zona glomerulosa of the adrenal gland. It is a metabolite of progesterone, and a precursor of the glucocorticoid corticosterone and the mineralocorticoid aldosterone. Neuroactive metabolites of DOC, including (3α,5α)-3,21-dihydroxypregnan-20-one (THDOC), positively modulate γ-aminobutyric acid type A (GABA_A_) receptors (22), and produce subjective effects that resemble ethanol as shown using drug discrimination (23). As GABA_A_ receptors are a key receptor mechanism underlying the subjective interoceptive response to ethanol in primates (24), circulating neuroactive precursors, such as DOC, could alter ethanol self-administration. In addition to modulation of ionotropic receptor systems such as GABA_A_, neuroactive steroid precursors may interact with ethanol self-administration through other receptor systems. For example, mineralocorticoid receptors in the basolateral amygdala have been implicated in decreased anxiety-like behavior and decreased corticosterone in rodents (25). Thus, circulating and brain concentrations of mineralocorticoids and glucocorticoids may alter temperament, such as anxiousness, or other characteristics contributing to the risk of heavy ethanol consumption.

In cynomolgus monkeys, circulating levels of DOC are altered by pharmacological challenges to the HPA axis that isolate anatomical contributions to the stress response. Specifically, DOC is increased by injections of naloxone (hypothalamic) and CRH (pituitary), suppressed by dexamethasone (hypothalamic, pituitary, adrenal), but unchanged by ACTH (adrenal) or ethanol [1.0 g/kg or 1.5 g/kg, i.g., blood ethanol concentration (BEC) up to 147 mg/dl (26);]. In a study using low stress, resting conditions (i.e., remaining in the home cage) and in the absence of challenges to the HPA axis (i.e., a basal state), plasma DOC in ethanol-naïve male rhesus monkeys was negatively correlated with average longitudinal BEC measures across more than 12 months of daily access to ethanol and water (27). Among five hormones measured at baseline and after more than 12 months of daily ethanol access, DOC and aldosterone distinguished heavy from non-heavy drinkers; aldosterone positively correlated with both water and ethanol intake but not with level of intoxication as represented by average BEC. These data suggest that resting levels of DOC may have a unique predictive (negative) relationship with drinking to intoxication. In our earlier studies, DOC after saline, CRH, naloxone, or ACTH challenges in ethanol-naïve cynomolgus monkeys was not predictive of future ethanol self-administration, though the capacity of dexamethasone to suppress DOC in these subjects was negatively correlated with heavy drinking (26).

To date, the effect of chronic ethanol self-administration on HPA axis regulation of DOC is unknown. The present study reports basal DOC and its HPA axis regulation by each level of the HPA axis throughout the course of ethanol induction and self-administration. We hypothesized that ethanol self-administration would decrease adrenal sensitivity leading to a disruption in HPA axis stimulation of circulating DOC.

## Materials and Methods

### Animals

Adult male cynomolgus monkeys (*n* = 10 *Macaca fascicularis*, 70–85 months old when schedule induction started) were individually housed in 76 cm × 60 cm × 70 cm stainless steel cages in an environment maintained at 21 ± 1°C, with 30–50% humidity and a 11:13-h light cycle (lights on, 7:00 a.m.). All monkeys were maintained in a positive caloric and fluid balance throughout the experiment, as previously reported (26). This study was approved by the Wake Forest University Animal Care and Use Committee and conducted in accordance with both the Wake Forest University Animal Care and Use Committee guidelines and the guidelines for the care and use of laboratory animal resources (Commission on Life Sciences, National Research Council, 1996; NIH Guide for the Care and Use of Laboratory Animals).

The monkeys were trained using positive reinforcement to comply with awake venipuncture to collect blood for the steroid assays, as described elsewhere (26). To administer the pharmacological challenges, the monkeys were trained to sit in a primate chair using positive reinforcement for insertion of a intravenous catheter and nasogastric gavage using an infant feeding tube (5 French, 1.7 × 381 mm). During training, tap water was administered in volumes approximating volumes for ethanol administration during testing. All challenges of the HPA axis, except for the dexamethasone challenge, were conducted in the primate chairs.

### Blood Sampling

Femoral blood samples were obtained with a 22-g × 1-inch Vacutainer needle and a 3-ml Vacutainer hematology tube (Becton Dickinson, Franklin Lakes, NJ, USA). All blood samples were stored on ice (approximately 15 min) until centrifuged (3,000 rpm, 15 min at 4°C, Model Allegra 21R, Beckman Coulter, Fullerton, CA, USA). Plasma samples (100-µl aliquots) were frozen at −80°C and stored in 2-ml microtubes until processing.

### Ethanol Self-administration

The procedure for schedule induction and maintenance of long-term ethanol self-administration is described in detail elsewhere (20, 28, 29). Briefly, monkeys were trained to operate a drinking panel (Med-Associates, Inc., St. Albans, VT, USA) attached to one wall of each monkey’s home cage. All fluid was available through two spouts activated by pulling a centrally located dowel, one for water and another for ethanol, and food requirements by pressing a push panel. Initially, fluid was available through the right-side drinking spout, and one press on the push panel resulted in presentation of a 1-g banana-flavored pellet (carbohydrate: 63%; fat: 4%; protein: 22%; PJ Noyes, Lancaster, NH, USA). Training was complete (approximately 2–3 weeks) once the monkey reliably pulled the dowel to activate the panel, drank from the spouts, and received all available food pellets by responding on the push panel. Subsequently, monkeys were induced to consume water under fixed-time pellet delivery to establish schedule-induced polydipsia (SIP). Monkeys were induced to drink 150–250 ml of water (corresponding to the volume necessary to self-administer an ethanol dose of 1.5 g/kg, with a concentration of 4% w/v) each day for 30 days. Monkeys were then induced to drink 0.5 g/kg of ethanol (4% w/v) per day for 30 consecutive days, 1.0 g/kg/day for 30 consecutive days, and finally 1.5 g/kg/day for 30 consecutive days. After schedule induction, all monkeys had 22 h/day open access to ethanol and water for approximately 6 months, and the daily allotment of food was available in three equal “meals” available at the start of the daily session and each separated by 2 h. Summaries of the ethanol self-administration data are available online (www.MATRR.com; cynomolgus cohort 1).

### Pharmacological Profiling of the HPA Axis

Each of six pharmacological challenges [naloxone, ovine CRH (oCRH), ACTH, dexamethasone, and ethanol (two doses)] was conducted at baseline, after schedule induction and after 6 months of ethanol access between 8:00 a.m. and 2:00 p.m. (lights on at 7:00 a.m.). Two challenges per week were conducted, requiring 4 weeks to complete all challenges: Week 1, dexamethasone (130 µg/kg, intramuscular) on Monday/Tuesday, ACTH (0.5 mg/kg intramuscular dexamethasone followed 4–6 h by 10 ng/kg intravenous Cortrosyn) on Thursday; Week 2, naloxone 375 µg/kg on Friday; Week 3, saline (0.35 ml intramuscular) on Tuesday, ethanol (1.0 g/kg, intragastric) on Friday; Week 4, ethanol (1.5 g/kg, intragastric) on Tuesday, CRH (1 µg/kg, intravenous) on Friday. The procedure for each challenge differed according to the challenge. For the dexamethasone suppression test, blood was obtained pre-dexamethasone (8:00 a.m.), dexamethasone was administered at 10:00 p.m. the same day, and the next morning (8:00 a.m.) another blood sample (post-dexamethasone) was obtained. For the ACTH challenge, the monkeys were administered dexamethasone after an overnight fast. Four to six hours later, a baseline blood sample was obtained and ACTH administered. Blood was obtained 15 and 30 min after ACTH infusion. After naloxone was administered, blood was collected at 15, 30, 60, 90, and 120 min. Blood samples were obtained 30 min before ethanol administration then 15, 60, 90, and 120 min after. For the ethanol challenges, BEC was measured from the 60, 90, and 120 min samples. For the CRH challenge, a baseline blood sample was collected 30-min prior to administration of oCRH and 15, 30, and 60 min after administration.

These studies were designed to allow adequate time between endocrine challenges. After five half-lives, a drug is effectively cleared from circulation. In the case of dexamethasone, which has the longest half-life of the drugs used in these studies (approximately 190 min in humans), this would be cleared in less than 16 h. Pharmacological challenges were separated by a minimum of 72 h. While the immediate effects of these pharmacological challenges would be gone, there is a possibility that they cause a longer lasting effect. To control for this, the challenges were performed in the same order at each experimental phase. In humans the half-life of the other drugs use are as follows: naloxone (64 ± 12 min), oCRH (43 ± 6 min), ACTH (intravenous, 15 min), and dexamethasone (190 min).

### Drugs

Naloxone hydrochloride dihydrate and oCRH were purchased from Sigma-Aldrich Co., St. Louis, MO, USA. ACTH (Cortrosyn; 0.25 mg/vial reconstituted with sterile saline; Amphastar Pharmaceuticals, Inc., Rancho Cucamonga, CA, USA) and dexamethasone (10 and 4 mg/ml for the ACTH and dexamethasone profiles, respectively; Baxter, Deerfield, IL, USA) were obtained in a commercial formulation from the Wake Forest University Baptist Medical Center pharmacy. All drugs were diluted in sterile saline to make the appropriate concentrations. Ethanol (95%, Warner-Graham, Cockeysville, MD, USA) was diluted in tap water to a concentration of 20% (w/v) for the 1.0 g/kg dose and 30% (w/v) for the 1.5 g/kg dose to keep volume constant across both doses.

### DOC Assay

All plasma samples were assayed for DOC using radioimmunoassay (RIA) as previously described (26). Briefly, plasma samples (200 µl) were extracted twice with 2 ml ethyl acetate/hexane (3:2); 1,000 cpm of [^3^H]DOC (SpA = 50 Ci/mmol; American Radiolabeled Chemicals, Inc., Saint Louis, MO, USA) were added to each sample for recovery estimation. The dried extracts were resuspended in 1.5 ml RIA buffer of which 0.5 ml were used for the assay (run in duplicate) and 0.3 ml were used for recovery determination. Each assay included a rat plasma sample as an internal control. The antiserum for DOC was purchased from MP Biomedicals (Solon, OH, USA) and diluted according to manufacturer’s instructions. This antiserum was highly specific for DOC as shown by the following cross-reactivity tests: DOC 100%, 3α,5α-THDOC 4.7%, progesterone 2.5%, corticosterone 1.7%. Less than 1% cross-reactivity was observed for (3α,5α)-3-hydroxypregnan-20-one (3α,5α-THP), 3α-hydroxy-pregn-4-en-20-one, pregnenolone, 20-hydroxy-pregnen-4,3-one, testosterone, androstenedione, 17α-hydroxyprogesterone, 11-deoxycortisol, 5α-dihydrotestosterone, cortisol, cholesterol, 17β-estradiol, estrone, and estriol. Unknown samples were compared to concurrently run standards using a one-site competition model and adjusted for extraction efficiency. DOC values are expressed as nanograms per milliliter of plasma. The sensitivity of the assay is 10 pg/ml. Intra-assay and inter-assay coefficients of variation were 4.86 and 4.51%, respectively. ACTH was assayed in duplicate at Yerkes Endocrine Core Laboratory (Atlanta, GA, USA) using a commercially available kit (DiaSorin kit #24130; Stillwater, MN, USA), with sensitivity of 6.8–436.0 pg/ml.

### Data Analysis

Area under the curve (AUC) was calculated using the trapezoidal method. Basal, AUC, and peak concentrations were log-transformed, when necessary, prior to statistical analysis. The effects of pharmacological challenges on plasma DOC were analyzed using linear mixed-effect models with monkey as the subject variable and experimental phase (three levels: baseline, induction, and self-administration) as the independent variables. Significant results are reported based on *post hoc* comparisons using Tukey-corrected *t*-tests. All analyses were conducted using R 2.15.3 (R Core Team, 2015), α < 0.05. All data are presented as mean ± SD unless stated otherwise.

## Results

### Basal DOC

**Figure 1:**
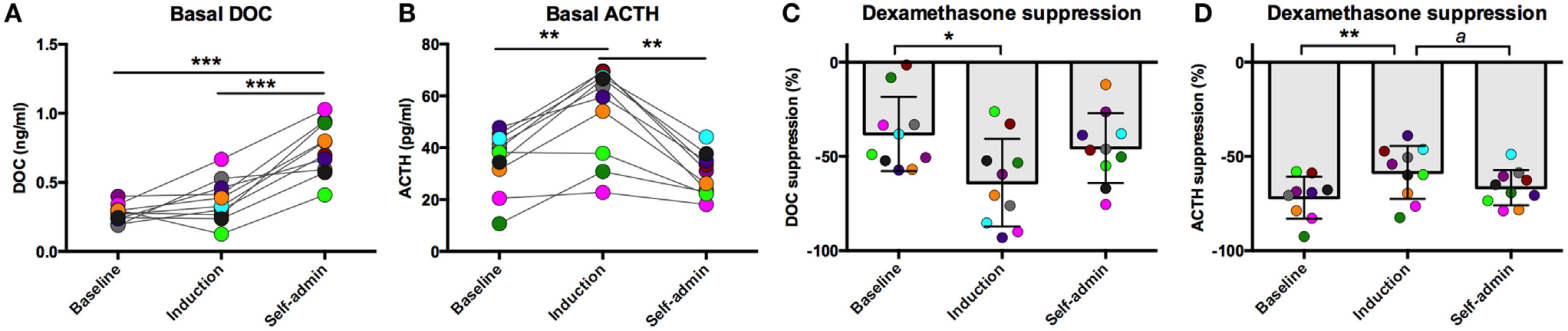
**Basal deoxycorticosterone (DOC) (A) and adrenocorticotropin hormone (ACTH) (B) measured from samples collected while animals were in their home cage in the absence of pharmacological challenge**. Dexamethasone suppression (percent of baseline) of DOC **(C)** and ACTH **(D)**. Animals are uniquely colored. ^a^*p* < 0.05, *p < 0.01, **p < 0.001, ***p < 0.0001.

All animals were trained to participate in awake venipuncture and had been doing so each week in their home cage for over 6 months prior to collection of the samples reported here. This protocol reliably produces low levels of circulating stress hormones (30, 31). Under this low stress condition, basal DOC increased over the course of the experiment [*F*(2,18) = 45.0, *p* < 0.0001, Figure 1A]. Compared to baseline (0.28 ± 0.06 ng/ml) and induction (0.37 ± 0.16 ng/ml), DOC had increased twofold following 6-months of ethanol self-administration (0.74 ± 0.19 ng/ml; *p* < 0.0001). This was intriguing, as we have previously reported a positive correlation between plasma ACTH and DOC (26) and that ACTH is elevated during ethanol induction (32). Given this relationship, we assayed ACTH from the same samples. As we have previously reported, ACTH varied by experimental phase [*F*(2,18) = 19.1, *p* < 0.0001, Figure 1B] and was highest during induction (54.2 ± 17.4 pg/ml) compared to baseline (35.3 ± 11.7 pg/ml; *p* < 0.0004) and 6 months self-administration (29.5 ± 8.2 pg/ml; *p* < 0.0004). Under induction, ACTH, but not cortisol, is elevated for prolonged periods of time (32). These data suggest that while DOC concentration is influenced by HPA axis activation, it is not directly linked to circulating ACTH.

During open access, the average daily consumption ranged from 1.2 to 4.2 g/kg/day, with a group average (mean ± SEM) of 2.7 ± 0.3 g/kg/day. Average daily consumption was not correlated with basal DOC [baseline: *r*(9) = −0.33, *p* = 0.36; induction: *r*(9) = −0.07, *p* = 0.85; 6 months: *r*(9) = 0.17, *p* = 0.64] or ACTH [baseline: *r*(9) = 0.04, *p* = 0.91; induction: *r*(9) = −0.34, *p* = 0.34; 6 months: *r*(9) = 0.06, *p* = 0.87].

### Dexamethasone Suppression Test

We have previously reported that DOC is modulated by the HPA axis in non-human primates (26). Additionally, an extensive literature describes the consequences of long-term alcohol consumption on HPA axis function. We aimed to further understand the interaction between HPA axis activation and DOC within subject across a well-validated model of ethanol self-administration. One well-documented consequence of HPA axis over-activation is decreased sensitivity to glucocorticoid negative feedback (10, 14). Dexamethasone is a synthetic glucocorticoid with high affinity for the glucocorticoid receptor and is used to measure sensitivity to negative feedback. Ethanol induction differentially influenced sensitivity to dexamethasone suppression for both DOC and ACTH [DOC: *F*(2,18) = 4.20, *p* = 0.032; ACTH: *F*(2,17) = 6.67, *p* = 0.007; Figures 1C,D]. DOC showed greater suppression during induction compared to baseline (*p* = 0.01), while ACTH was less sensitive to dexamethasone suppression during induction when compared to both baseline and self-administration (*p* = 0.001 and *p* = 0.038, respectively).

### Post-Dexamethasone ACTH Challenge

**Figure 2:**
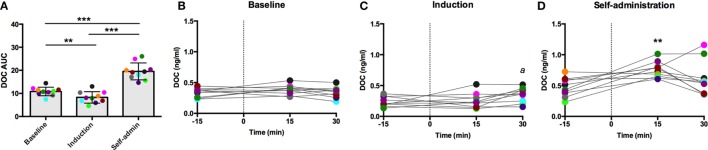
**Exogenous ACTH (10 ng/kg, 4–6 h after 0.5 mg/kg dexamethasone) stimulation of circulating deoxycorticosterone (DOC)**. DOC response is summarized across the experimental phases by area under the curve **(A)**. DOC concentrations for each timepoint sampled during baseline **(B)**, induction **(C)**, and self-administration **(D)** are shown. Symbols represent significant differences when compared to baseline (*t* = −15min). Animals are uniquely colored. ^a^*p* < 0.05, **p < 0.001, ***p < 0.0001.

Each challenge had unique sample collection times, which are indicated on the *x*-axis of their respective figures. Dexamethasone suppresses adrenal and pituitary secretions allowing for the isolated adrenal response to an exogenous ACTH to be measured. DOC sensitivity, assessed by AUC, varied across the experimental phases [*F*(2,18) = 63.28, *p* < 0.0001; Figure 2A]. In line with our hypothesis of decreased adrenal sensitivity during ethanol induction, DOC AUC was significantly blunted during this phase compared to baseline (*p* = 0.0006). Following self-administration, DOC AUC was significantly greater than both baseline and induction (*p* < 0.0001), supporting heightened adrenal sensitivity to ACTH. Post-dexamethasone ACTH was generally found to be a weak stimulus for DOC secretion in these animals, as shown in the individual timecourses (Figures 2B–D). At baseline, there was no main effect of time [*F*(2,18) = 1.3, *p* = 0.30; Figure 2B], indicating DOC concentrations remained flat following the challenge. During induction, a moderate increase was seen [*F*(2,18) = 3.7, *p* = 0.05; Figure 2C], where DOC concentration 30 min after the challenge (0.35 ± 0.12 ng/ml) was significantly higher compared to pre-challenge (*t* = −30; 0.24 ± 0.08 ng/ml). Following 6 months of ethanol self-administration, DOC showed an increased sensitivity to ACTH challenge [*F*(2,18) = 61.8, *p* < 0.0001; Figure 2D]. DOC concentrations peaked at *t* = 15 min (0.76 ± 0.13 ng/ml) and were significantly greater than baseline (*p* < 0.0001). These results should be considered within the context of basal DOC levels (shown in Figure 1). Although the increase over the timecourse was found to be significant during both induction and self-administration, this is largely due to the low pre-challenge concentration as the peak concentration is not above basal levels at either experimental phase. The response to this challenge is in agreement with the results of basal DOC and supports our hypothesis of dampened adrenal sensitivity during ethanol induction and enhanced sensitivity during self-administration.

### Exogenous oCRH Administration

**Figure 3:**
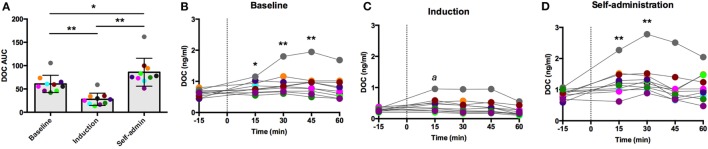
**Deoxycorticosterone (DOC) response to exogenous ovine CRH (1.0 µg/kg) administration**. The DOC response is summarized across the experimental phases by area under the curve **(A)**. DOC concentrations for each timepoint sampled during baseline **(B)**, induction **(C)**, and self-administration **(D)** are shown. Symbols represent significant differences when compared to baseline (*t* = −15min). Animals are uniquely colored. ^a^*p* < 0.05, *p < 0.01, **p < 0.001.

Intravenous oCRH does not cross the blood–brain barrier, thus stimulating pituitary synthesis and secretion of ACTH without stimulating contribution from the hypothalamus. As shown in Figure 3A, the AUC response to oCRH varied by experimental phase [*F*(2,18) = 119.66, *p* < 0.0001]. Similar to post-dexamethasone ACTH, DOC AUC during induction was significantly reduced compared to baseline and self-administration (*p* < 0.001), and the AUC was significantly higher during self-administration when compared to baseline (*p* = 0.002). Prior to ethanol, peak DOC concentration in response to the oCRH challenge was 0.92 ± 0.40 ng/ml at *t* = 45 min (Figure 3B). Peak DOC was significantly dampened during ethanol induction (0.44 ± 0.22 ng/ml, *t* = 15 min; Figure 3C), and a heightened response was found during self-administration (1.36 ± 0.54 ng/ml, *t* = 30 min; Figure 3D). These results provide further evidence of dampened adrenocortical DOC response to ACTH stimulation during induction and a compensatory over-sensitization following self-administration.

### Naloxone Challenge

**Figure 4:**
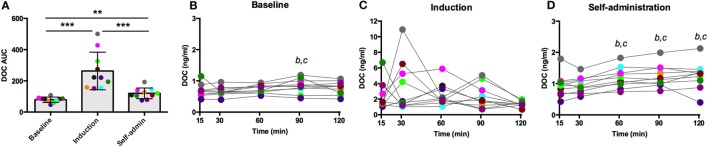
**Deoxycorticosterone (DOC) response to naloxone (375 µg/kg) administration**. The DOC response is summarized across the experimental phases by area under the curve **(A)**. DOC concentrations for each timepoint sampled during baseline **(B)**, induction **(C)**, and self-administration **(D)** are shown. Symbols represent significant differences when compared to *t* = 15 (*b*) or *t* = 30 (*c*); see main text for specific *p*-values. Animals are uniquely colored. **p < 0.001, ***p < 0.0001.

Naloxone is a competitive opioid antagonist that releases the inhibitory tone on the HPA axis, stimulating synthesis and release of ACTH and adrenal steroids. As summarized in Figure 4A, DOC AUC varied across the experimental phases [*F*(2,18) = 55.61, *p* < 0.0001]. Compared to baseline, ethanol induction greatly potentiated the effect of naloxone (*p* < 0.0001), which was reduced during self-administration (*p* < 0.0001, compared to induction), although in this latest phase it remained significantly elevated compared to baseline (*p* = 0.0006). Across the sampling timecourse, naloxone resulted in a moderate increase in DOC at baseline [*F*(4,36) = 4.13, *p* = 0.007, Figure 4B]. Peak DOC concentration measured at *t* = 90 min (0.83 ± 0.23 ng/ml) was significantly higher compared to *t* = 15 (0.68 ± 0.20 ng/ml; *p* = 0.01) and *t* = 30 (0.67 ± 0.14 ng/ml; *p* = 0.008). During induction, there was a wide range in circulating DOC concentration across all timepoints that obscured any main effects of time [*F*(4,36) = 1.46, *p* = 0.23, Figure 4C, notice the scale of the *y*-axis]. Following 6 months of ethanol self-administration, a significant effect of time was found [*F*(4,36) = 7.62, *p* < 0.0001, Figure 4D], and the peak concentration was measured at *t* = 120 min (1.3 ± 0.4 ng/ml). These results suggest central mechanisms mediating HPA axis activity are under greater inhibitory control during ethanol induction. Such an increase in inhibitory opioid tone at the level of the hypothalamus may compensate for the decreased sensitivity of ACTH to glucocorticoid negative feedback.

The responses to the pharmacological challenges described so far were not found to be predictive or dependent on average daily ethanol intake, thus the specific effects of ethanol are unclear. To address this, animals were challenged with two doses of intragastric ethanol (1.0 and 1.5 g/kg).

### Ethanol Challenge

**Figure 5:**
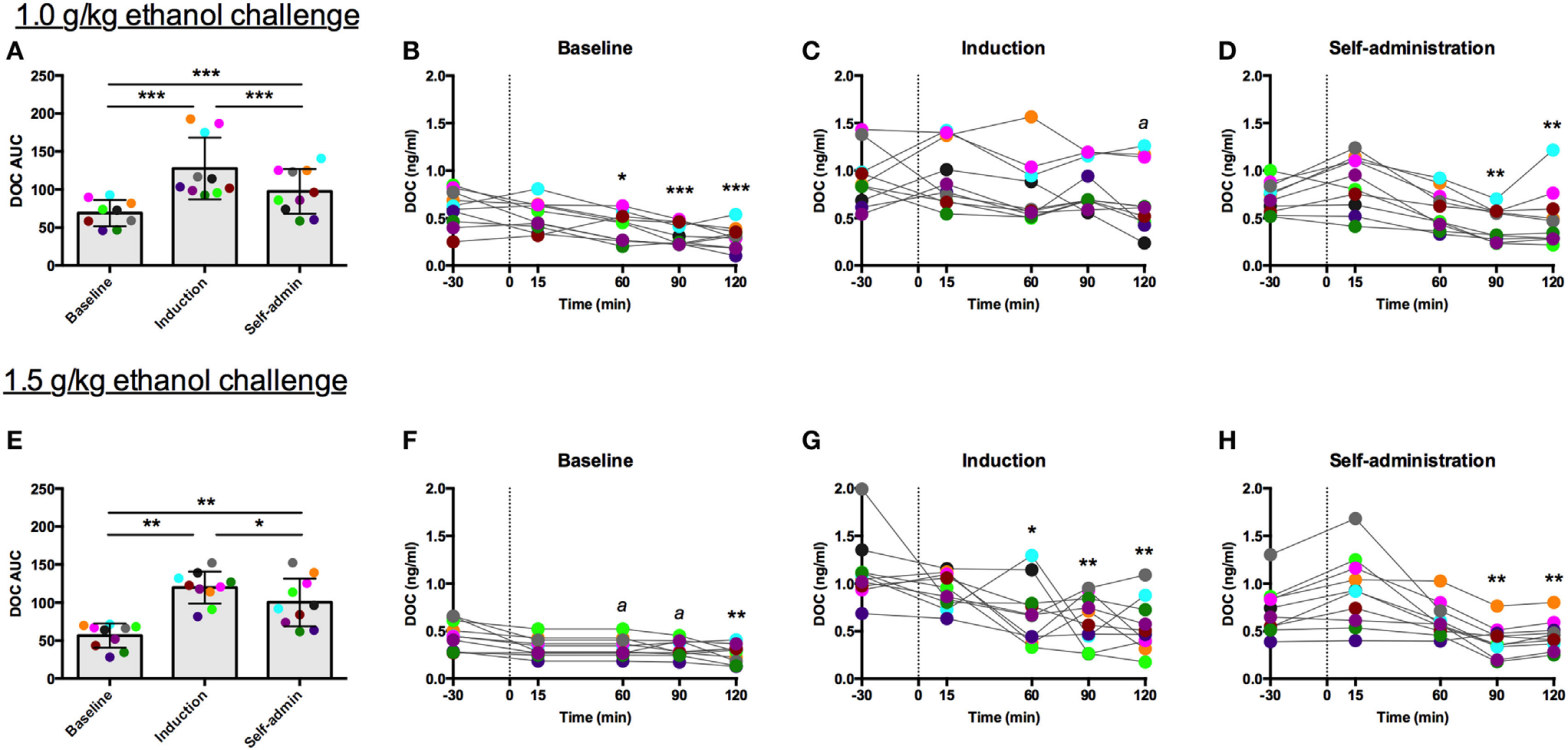
**Deoxycorticosterone (DOC) response to 1.0 and 1.5 g/kg oral ethanol gavage**. DOC response is summarized across the experimental phases by area under the curve [**(A,E)** respectively]. DOC concentrations for each timepoint sampled during baseline **(B,F)**, induction **(C,G)**, and self-administration **(D,H)** are shown. Symbols represent significant differences when compared to baseline (*t* = −30 min). Animals are uniquely colored. ^a^*p* < 0.05, *p < 0.01, **p < 0.001, ***p < 0.0001.

For both challenge doses, there was a main effect of time [1.0 g/kg: *F*(2,18) = 51.85, *p* < 0.0001, Figure 5A; 1.5 g/kg: *F*(2,18) = 44.09, *p* < 0.0001, Figure 5E]. DOC AUC was significantly higher during induction compared to baseline (1.0 g/kg: *p* < 0.00001; 1.5 g/kg: *p* < 0.001) and self-administration (1.0 g/kg: *p* < 0.001; 1.5 g/kg: *p* = 0.04). Although the AUC was lower during self-administration when compared to induction, it was still significantly elevated when compared to baseline (1.0 g/kg: *p* < 0.00001; 1.5 g/kg: *p* < 0.001). While these two challenge doses resulted in similar AUC across the experimental phases, average BEC differed between the challenges but were consistent across the experimental phases (1.0 g/kg: baseline: 92 ± 13 mg/dl, induction: 92 ± 20 mg/dl, self-administration: 101 ± 20 mg/dl; 1.5 g/kg: baseline: 149 ± 17 mg/dl, induction: 162 ± 19 mg/dl, self-administration: 168 ± 26 mg/dl). These data indicate that metabolic tolerance is an unlikely source of any of the changes reported in these animals.

The individual timecourses show that both challenge doses of ethanol blunted circulating DOC. This effect was less robust during induction, as indicated by the rise in AUC, where percent reduction went from 50 to 22% following 1.0 g/kg (Figure 5C) and 41 to 12% following 1.5 g/kg (Figure 5G). During both baseline and induction the lowest DOC concentration was measured at 120 min following the challenge. Following ethanol self-administration, DOC suppression normalized, 31% for 1.0 g/kg (Figure 5D) and 43% for 1.5 g/kg (Figure 5H). Thus, while direct activation of the HPA axis by the other pharmacological challenges resulted in an increase in circulating DOC, ethanol challenges produced the opposite effect. Similar to the naloxone challenge, AUC was elevated during induction, an effect primarily due to the elevated baseline DOC concentrations.

## Discussion

The current dataset is an extensive investigation of the mechanisms related to HPA axis activation that regulate DOC concentration over the course of ethanol induction and self-administration in non-human primates. In these animals, trained to participate in awake blood collection, the traditional circulating stress hormones (ACTH and cortisol) are low and have a diurnal rhythm indicating low basal stress (30, 31). Thus, our blood collection procedure provides a stable condition under which the native circuitry regulating the adrenal output appears to be in homeostasis at baseline and can serve as a comparative basis across the experimental phases. Furthermore, the state of the HPA axis within a phase of ethanol self-administration appears reliable as evidenced by elevated ACTH concentrations during ethanol induction, a finding we have previously reported in different samples from the same animals (32).

Circulating DOC in human and non-human primates is mainly of adrenal origin, and its secretion is regulated by the HPA axis, in a manner similar to cortisol (26, 33–36). The data presented here further support a regulation of circulating DOC levels by the HPA axis under baseline conditions and following long-term ethanol exposure in cynomolgus monkeys. Overall, it appears that HPA axis regulation and feedback are disrupted by ethanol consumption under SIP parameters. This outcome is consistent with data showing schedules of reinforcement that generate SIP rely on establishing a conflict between aversive and appetitive aspects of the schedule (37). Once the induction schedule is removed and the monkey is given 22 h/day (open access) to choose to drink ethanol or water, there is an immediate decrease in cortisol (32) and, by inference, a likely decrease in DOC. After 6 months of daily open access ethanol self-administration, the DOC responses to the HPA axis challenges are lower than during ethanol induction, although uniformly higher than baseline, suggesting that chronic ethanol self-administration results in a new allostatic state.

The large influence of the SIP condition is evidenced by the adaptation to pharmacological challenge between baseline and induction. These divergent, phase-dependent responses indicate a shift in HPA axis regulation of DOC that may be attributed to ethanol, the scheduled induction, or their combination. These data demonstrate that the adrenal cortex, specifically the zona fasciculata, is less sensitive to ACTH stimulation (Figures 2 and 3) but more sensitive to hypothalamic disinhibition (naloxone challenge, Figure 4). The heightened DOC response to naloxone during induction could indicate greater opioid receptor occupancy, as cortisol response to naloxone predicted μ-opioid receptor binding potential in positron-emission tomography studies of the hypothalamus, caudate, putamen, and ventral striatum of humans (38). From a homeostatic point of view, the heightened opioid control at the level of the hypothalamus could be a compensatory change established to rebalance control over HPA axis activity in response to dampened adrenal sensitivity to ACTH, resulting in an allostatic state (39). However, further studies are needed to address this hypothesis.

The development of an allostatic state following the ethanol self-administration protocol is clearly supported by the changes in basal (unchallenged, Figure 1A) DOC response across the experimental phases. Despite aberrant responses to naloxone and exogenous ACTH during induction, no differences were found in basal DOC between baseline and induction. However, following 6 months of daily open access ethanol self-administration, when the responses to pharmacological challenges had moved toward their baseline levels of responding, basal circulating DOC became significantly higher than during either the preceding baseline or induction phases. Furthermore, although DOC remained reliably suppressed by the administration of 1.5 g/kg ethanol following prolonged self-administration, the suppression was delayed compared to the initial (baseline) ethanol response. Thus, it appears that chronic ethanol consumption may increase circulating DOC and thereby the production of its neuroactive metabolites. In humans, any increase in basal DOC may be reversible as it has been reported that 1-month-abstinent alcoholics and controls had similar DOC, ACTH, progesterone, 17-OH-progesterone, aldosterone, androstenedione, DHEA, and pregnenolone sulfate levels (10, 36).

Importantly, the data presented here help characterize the circumstances of HPA axis activation of DOC secretion. Specifically, a dysregulation between HPA axis activation and DOC secretion at each level of the HPA axis was revealed, particularly during induction but also during chronic ethanol self-administration. In addition to the difference in 1-month-abstinent alcoholics cited above, these data are in contrast to a study that found ethanol administration for 3 weeks did not change basal DOC levels in the plasma or brain of rats (40). However, rats that received chronic (3 week) ethanol administration had decreased levels of corticosterone (41) and blunted ethanol-induced elevations of cerebral cortical 3α,5α-THP and DOC levels (40, 42). The apparent discrepancies in regulation of DOC responses following long-term ethanol exposure might be the result of species differences or the different experimental procedures, i.e., assessment immediately following relatively short term (3 weeks) alcohol exposure in rats (40) or long term (over 9 months of ethanol) in cynomolgus monkeys (present results) versus 1-month abstinence with a highly variable history of alcohol in humans (36). The results presented here support the need for a greater mechanistic understanding of ethanol-induced changes in steroidogenic pathways within the adrenal cortex, as well as the PVN and the specific contribution of the opioid receptors to the changes reported here.

It is important to consider the potential contribution of hormonal rhythms in the current results. The pharmacological challenges and blood samples were collected at the same time of day across the experimental timeline, but the seasonal contributions were not controlled. Adrenal and pituitary hormones are known to have annual rhythms that are maintained under laboratory conditions in rhesus males (43). In humans, peak cortisol was reportedly higher in the winter compared to summer (44). Therefore, DOC concentration may also follow a seasonal pattern and be higher in the winter. However, both the baseline and 6-month self-administration challenges were performed during the winter (January and February of consecutive years, respectively), while greatest DOC response was found following induction of ethanol, when seasonal rhythms would have predicted a lower response. Thus, seasonal differences in adrenal sensitivity do not appear to have contributed to the DOC responses reported here.

Overall, the hormone-specific effects of ethanol in macaques suggest unique effects in different zones of the adrenal cortex that require further study. As a first step, the present data show differential effects of pre-ethanol, ethanol induction, and voluntary ethanol self-administration on opioid, ACTH, and glucocorticoid regulation of circulating DOC levels. These results have implications for neurosteroid activity in the brain, and we have shown that chronic ethanol self-administration protocol reduces 3α,5α-THP in the amygdala (45). Furthermore, because the greatest adaptations in DOC response were found with opioid regulation of the HPA axis, it appears that circulating DOC is a reflection of chronic ethanol-induced long-term allostasis involving central mechanisms. However, future studies will need to better define the mechanism(s) supporting an allostatic state such as altered synthesis, release or degradation of DOC as well as altered pharmacodynamics of the opioid, ACTH, and glucocorticoid receptors that help regulate HPA axis activity.

## Author Contributions

KG designed the experiments and oversaw all aspects of data collection, analysis, and interpretation. PP and AM performed the DOC assays. VJ performed the analyses. All the authors contributed to planning the analyses and preparing the manuscript.

## Conflict of Interest Statement

The authors declare that the research was conducted in the absence of any commercial or financial relationships that could be construed as a potential conflict of interest.
